# Tribological Studies of Bamboo Fibre Reinforced Epoxy Composites Using a BOD Technique

**DOI:** 10.3390/polym13152444

**Published:** 2021-07-25

**Authors:** Ayedh Eid Alajmi, Jasem Gh. Alotaibi, B. F. Yousif, Umar Nirmal

**Affiliations:** 1 Department of Automotive and Marine Engineering Technology, Public Authority for Applied Education and Training, Adailiyah 122001, Kuwait; ae.alajmi@paaet.edu.kw (A.E.A.); jg.alotaibi@paaet.edu.kw (J.G.A.); 2 Faculty of Health, Engineering and Sciences, The University Southern Queensland, Toowoomba, QLD 4350, Australia; 3 Centre of Advanced Mechanical and Green Technology, Faculty of Engineering and Technology, Multimedia University, Jalan Ayer Keroh Lama, Melaka 75450, Malaysia; nirmal@mmu.edu.my

**Keywords:** composites, friction and wear behaviour, tribological properties, reinforcement

## Abstract

To reduce the emission of harmful materials into the ecosystem, researchers have been exploring the potential of manufacturing polymeric composites based on natural fibres. Although the large area of application of these materials has encouraged investigations of their performance under various loading conditions, less research has been conducted on their tribological behaviour. Hence, in this study, tribological tests were conducted on epoxy composites based on bamboo fibres. The wear performance of bamboo fibre reinforced epoxy was tested using various operating parameters, and the worn surfaces were examined using optical microscopy. The results revealed that the specific wear rate of the composites reduced since the epoxy was reinforced with bamboo fibres. Scanning electron microscopy analysis showed different wear mechanisms and damages.

## 1. Introduction

In the past decade, we had witness openly major destruction caused by mankind to the nature. Forest being torn down for housing developments projects, high rise buildings being build one after another to showcase the technology and the pride of one’s country, burning of harvested agricultural waste openly to discard it in a fast manner without considering the side effects to the environment, huge piles of garbage piled up in landfill areas as a result of unnecessary garbage produced from homes, disposal of hazardous material to the environment due to lack of awareness and stringent policies; and the list goes on and on. It is known that countries located at the equatorial region are blessed with the high growth rate of agriculture plants which results in the production of huge amount of harvested agricultural waste. Table 1 summarizes the non-useful bioproduct derived from the different types of agriculture plants.

Over time, mankind has realized that there is a crucial need for a balanced ecosystem between flora and fauna that can provide benefits. Although much damage to the earth has been done, efforts in research and development pertaining to the aforesaid matter have begun in earnest. At present, there is much attention paid to using natural fibres derived from the production of agriculture waste as potential reinforcements to form low-cost composite materials for a various applications. The term “cellulosic era” had been used widely by researchers due to the enormous attention paid to natural fibres. 

Taking bamboo fibres for instance, there is lack of information reported on its tribological wear performance when used as reinforcement with epoxy resin. For an instance, Tong and co-researchers [1] studied the wear volume of pure bamboo fibres. They reported that that the wear behaviour of bamboo fibres was a function of applied normal load, sliding velocity and orientation of fibres with respect to the sliding direction. Wear volume was superior when the fibres were orientated anti-parallel to the sliding counterface. The authors claimed that bamboo fibres had high resistance of material removal process due to the support received by the outer surface layer. The outer surfaces protected the inner soft surface from further delamination throughout the test. The predominant wear mechanisms were found to be micro-cracking and adhesion.

**Table 1:** Types of agriculture plants illustrating their useful and non-useful bioproducts and common method of disposal.

Plant	Useful Bioproduct Harvested	Non-Useful Bioproduct Disposed	Common Method of Disposal	References
Bamboo	Bamboo stripes for making furniture’s and souvenirs	Harvested bamboo trees and their leftover fibre stripes	Open burning, plant soil topping (i.e., plant fertilizers) burying, dumping at the sea	[1,2,3]
Betelnut	Betelnut seeds for traditional medicines	Fibre husks		[4,5,6]
Oil Palm	Oil palm fruits for making cooking oil	Oil palm fibre husks, and harvested oil palm trees		[7,8,9]
Kenaf	Kenaf fibre for making craft material such as food containers and souvenirs	‘over-matured’ kenaf fibres and harvested kenaf plants		[10,11,12,13]
Sugarcane	Sugarcane plant for juice consumption by humans	Sugarcane fibre husks		[14,15]
Coconut	Coconut fruit for juice consumption and cooking application	Coconut Hull’s and its fibre husks		[16,17]
Pineapple	Pineapple fruit for human consumption	Pineapple fibre husks derived from the fruit and leave		[18,19,20,21]

Preeti and co-researchers [22] reported on the usage of bamboo fibres along with other natural fibres to form a hybrid composite subjected to wear in sea water aging. They found that the composite had the tendency to absorb high amount of sea water due to its hydrophilic features observed in bamboo fibres. However, the authors claimed that the hybrid composite containing bamboo fibres showed improved wear properties as compared to other natural fibre composites. This is due to the structure of the bamboo fibre’s wall (i.e., contain a thin layer of wax like membrane) which prevents excessive absorption of sea water during the aging period.

In a work done by Shuna Chen et al. [23], Al_2_O_3_/rGO fibrous monolithic ceramics with bamboo-like architectures were used to study the tribological behaviour of the test samples. The authors claimed that the samples exhibited excellent fracture-resistant behaviours and high structural reliability. They revealed that moderate tribological behaviours with lower and more stable friction coefficients and wear rates is attainable when compared with monolithic Al_2_O_3_ ceramic. Further than that, the work concluded that bamboo fibre cell diameter directly influences the wear performance of the samples. 

From the above, it is obvious that bamboo fibre composites reveal convincing results in its wear and friction performance. Hence, the current work is an attempt to use bamboo stripes reinforced with epoxy resin for tribo-loading conditions. The test will be conducted on an indigenous Block on Disc (BOD) wear test rig where the test samples will be subjected to a smooth stainless steel counterface (i.e., SUS-304) to determine its optimum tribological characteristics. Different operating parameters such as applied normal loads: 10–32 N, sliding speeds: 1–4 m/s and sliding distance: 2.5–11 km will be considered. In addition, the surface roughness of the test samples and neat epoxy will be investigated to determine the abrasive effects to the stainless steel counterface. The morphology of the worn surface will be investigated using a digital high magnification microscope to determine the predominant wear mechanism. 

## 2. Methodology

### 2.1. Preparation of the Bamboo Fibres

**Figure 1:**
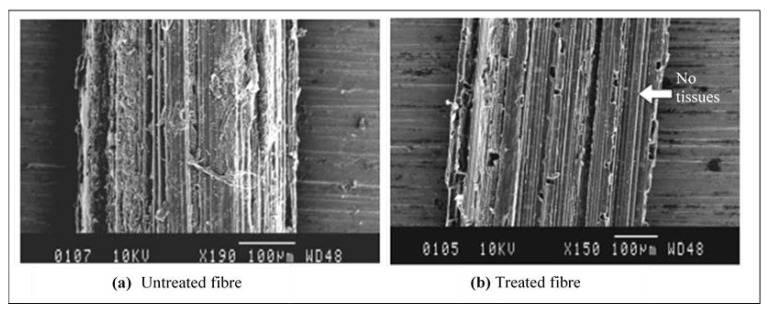
(**a**) Untreated and (**b**) treated fibre strips.

**Figure 2:**
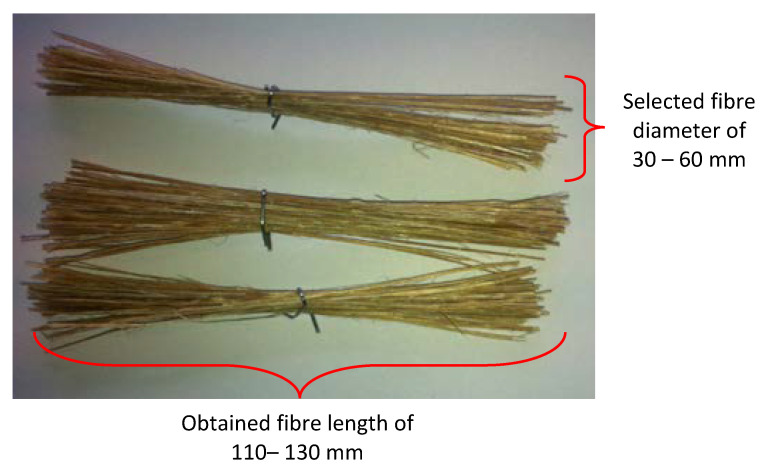
Selected stalked bamboo fibre strips for composite fabrication.

The bamboo fibre for this study was extracted using a manual method, given that some researchers have proved that manually extracted fibres show increased fibre density properties than do chemically extracted fibres. The node parts and the thin layers of bamboo bark were cleaned manually, and only the cylinder culm that contains the bamboo fibres was retained. Later, the cylinder culm was peeled down to obtain strips of different lengths and diameters. All strips were than soaked in 6% Sodium Hydroxide solution to treat the fibres for a duration of 24 h. The main idea here is to remove the lignin layer on the outer fibre surfaces as it acts as a wax thereby lowering surface wettability of the fibre/resin. Then, the fibres were taken out from the treatment and rinsed with distilled water to remove the excess impurities trapped within the fibres and remaining sodium hydroxide. It should be noted here that tap water is not used at this stage as it contains chlorine which can react with sodium hydroxide and modify the chemical properties of the bamboo fibres. Figure 1 shows the significant effects before and after treating the bamboo fibres with 6% NaOH. From the figure, it can be seen that the outer layer of the bamboo fibre surface is rougher after the treatment. This is a crucial factor during the dry sliding wear tribological test since the possibility of fibre to detach from the resin due to the sliding wear is low; i.e., good fibre/resin interfacial adhesion strength. The treated bamboo fibre strips were than left to dry at room temperature at 28 ± 5 °C for 24 h. When the fibres were completely dried, they were carefully selected under a 10x NK Vision microscope for diameters between 30–60 mm; Figure 2. This was done to ensure the homogenous dispersion of fibres during the fabrication process. All fibres had been post cured in an oven for 5 h at 45 °C and at a humidity level of 80 ± 10% to ensure proper drying of fibres and to enhance surface wettability of the fibres with the epoxy matrix. More information on the bamboo fibres extraction process is available at [2]. The density of the bamboo fibres was determined to be about 910 kg/m^3^, respectively. Table 2 illustrates some important information on the chemical and physical properties of the harvested bamboo fibres [21,22]. Table 3 presents the mechanical properties of the bamboo fibre and bamboo fibre/epoxy resin at different operating parameters [23,24].

### 2.2. Preparation of the Composite

A close steel mould of 100 × 100 × 15 mm^3^ was used for the purpose of fabricating the composite. The inner surface of the aluminium mould was sprayed with a thin layer of SPMRA (semi-permanent mould release agent). Dow Epoxy Resin (DER) 331 was used for this study because of its superior mechanical properties, such as low density, good strength, high chemical resistance and good adhesiveness. The resin mixed with hardener (50 wt%) were uniformly mixed with an electric stirrer (model: Braun AG, type 4-172) and poured slowly into the mould such that it formed a uniform thin layer of resin. Upon that, the treated bamboo fibres were uniformly placed on the layer of the epoxy resin as evenly as possible. This was achieved my means of a steel roller used to distribute the fibres evenly in the resin and to eliminate the generation of voids/air bubbles. Subsequently, another thin layer of epoxy resin mixed with 50 wt% of hardener was poured on the bamboo fibres, respectively. This process was repeated until the mould thickness of 15 mm was achieved. When the mould was completely filled with the mixture, a thin steel plate was placed on top of the mould’s opening. A pressure of about 5 kPa was applied on the steel plate to ensure that the trapped air bubbles in the composite were completely forced out. With the pressure still being applied on the mould, the composite block was left to cure for 24 h at room temperature (28 ± 5 °C). For thoroughness in curing, the hardened composite was removed from the mould and post-cured in an oven at 60 °C for one hour. The same procedure was repeated to fabricate neat epoxy (i.e., without the presence of bamboo fibres).

**Figure 3:**
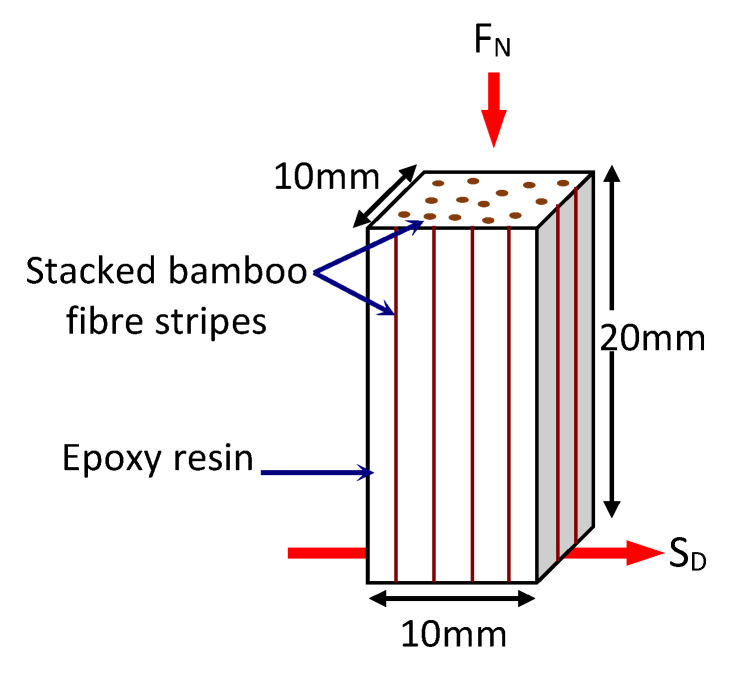
Schematic illustration of the Stalked Bamboo Fibre Reinforced Epoxy (S-BFRE) composite test specimen. Note: FN—Applied Normal Load; SDSliding Direction.

**Figure 4:**
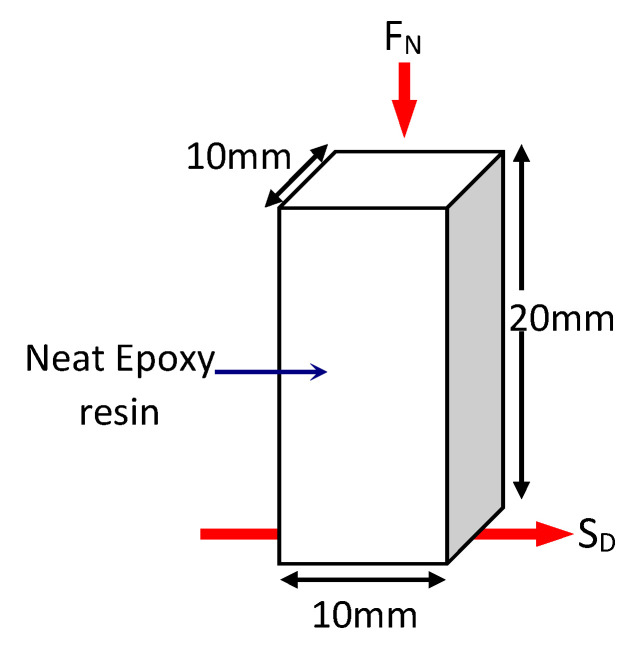
Schematic illustration of the Neat Epoxy (NE) test specimen.

For the purpose of conducting the tribological tests, test specimens with dimensions of 10 mm × 10 mm × 20 mm had been prepared from the cured composite block using a Black and Decker jigsaw (Model: CD301-B1). A schematic view of the tribological test specimen showing its fibre distribution is displayed in Figure 3 while the test specimens for neat epoxy is showed in Figure 4, respectively. The density of the composite was determined to be 1169.2 kg/m^3^.

### 2.3. Operational Parameters

Many studies have examined the tribological behaviour of natural fibres. Parameters such as speed, load and time that were used in this study were adopted from studies that recommended using these parameters for examining the tribology of natural fibres. Thus, NE specimens and bamboo/epoxy specimens were subjected to the same parameters to determine the differences in their performance. Table 4 lists the parameters used for the current work.

## 3. Experimental Procedure

**Figure 5:**
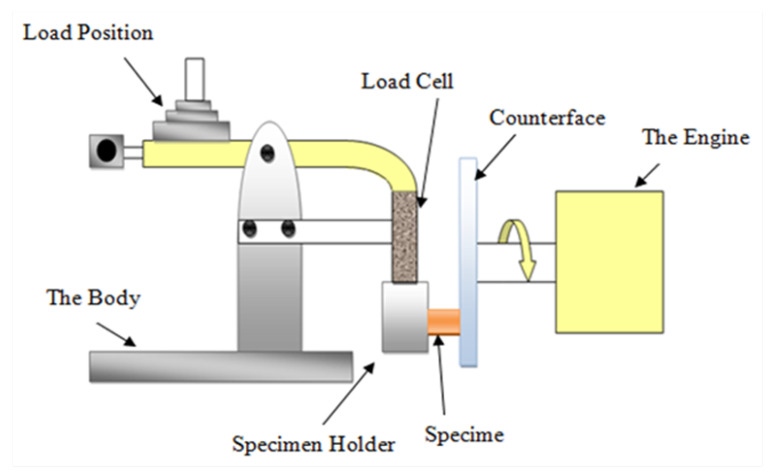
Tribology machine—block on desk configuration.

A tribological test on neat epoxy (NE) and bamboo/epoxy composites was conducted using a universal tribology machine at University of Southern Queensland, Figure 5. The experiments were conducted on block on desk configuration. Load cell was fixed on the arm of the configuration to measure frictional force between the rubbing surfaces and a digital friction sensor was used to capture frictional readings every second with the aid of data acquisition system. Before conducting the test, prepared samples and the counterface surface were polished using sand paper with grade of 500G–2000G to ensure the intimate contact between the sample’s surface and the counterface surface. The specimen was fixed in the specimen holder, and then the desired speed and load were set using the control panel. In the tribology software application, a thermo-image camera with infrared thermometer were used to measure the interfacial temperature between the specimen and the counterface during the test. Adhesive dry sliding tests were conducted at room temperature (25 °C) and under different applied loads as well as different velocities. Examination of the warm surfaces of all specimens was conducted using a microscope after each test. A Mahr Perthometer device was used to measure the roughness profile of each sample to identify the effect of different parameters in terms of roughness values.

All specimens were weighed before and after each test using a very sensitive scale. The specific wear rate (SWR) was calculated using Equation (1):

(1) $$Ws=\frac{\Delta V}{FnD}$$

where:

Ws = specific wear rate (mm^3^/N m)
ΔV = volume difference (mm^3^)
Fn = normal applied load
D = sliding distance (m)

The friction coefficient was determined using Equation (2). The same procedure was used for each specimen and the same measurements were taken.

(2) $$\mu =\frac{measured\;firctional\;force}{normal\;applied\;load\;}$$

## 4. Results and Discussion

**Figure 6:**
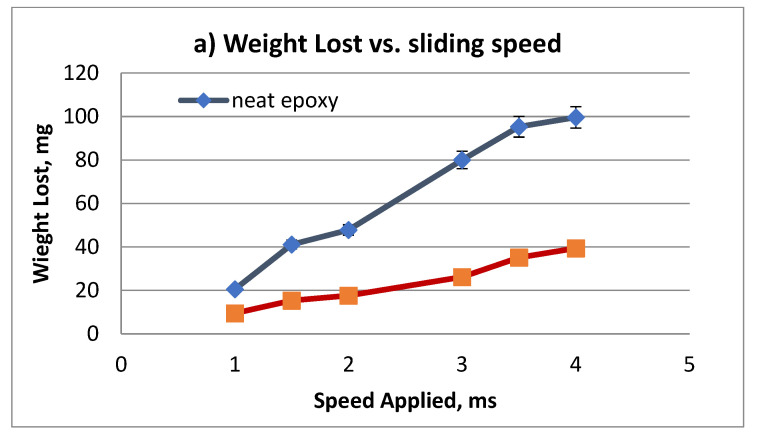

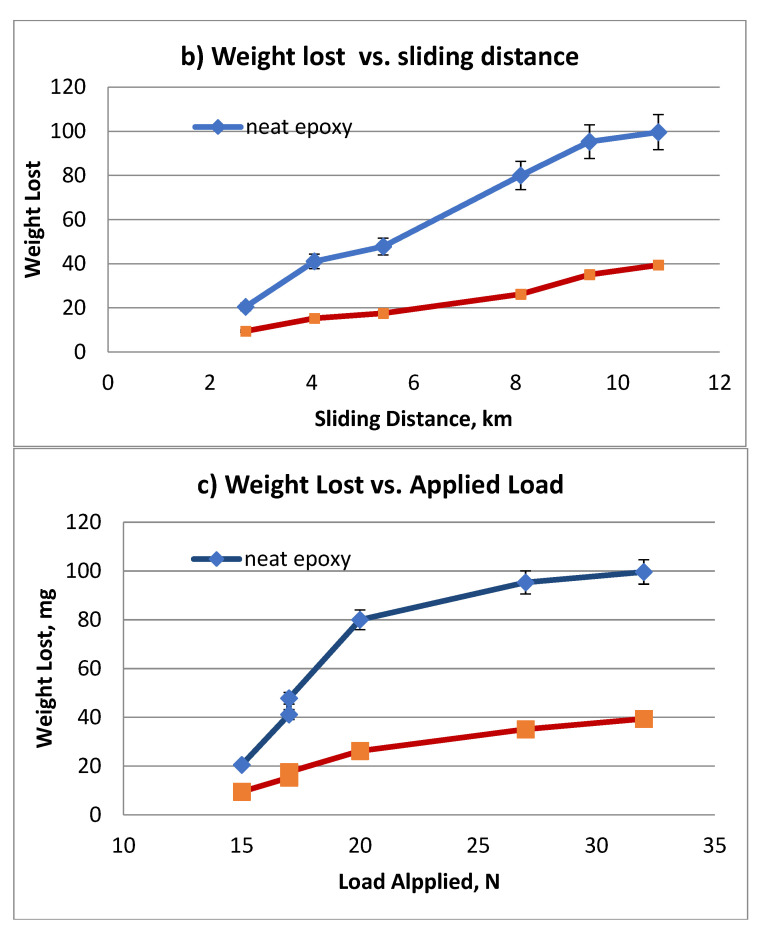
Relationship between weight lost and sliding distance, sliding speed and applied load.

The wear performance of NE and the bamboo fibre reinforced epoxy (S-BFRE) as a function of weight lost vs. sliding distance at different counterface sliding velocities subjected to different applied normal loads is shown in Figure 6. The figure reveals that the weight lost is significantly sensitive to the sliding speed as well to the applied load (i.e., the weight lost increased as the speed or the load increased). In detail, the increase in weight lost for the NE is entirely more because of increasing speed, load or sliding distance. This result shows that reinforcing epoxy with bamboo fibre can improve the weight lost performance of the polymer composite.

**Figure 7:**
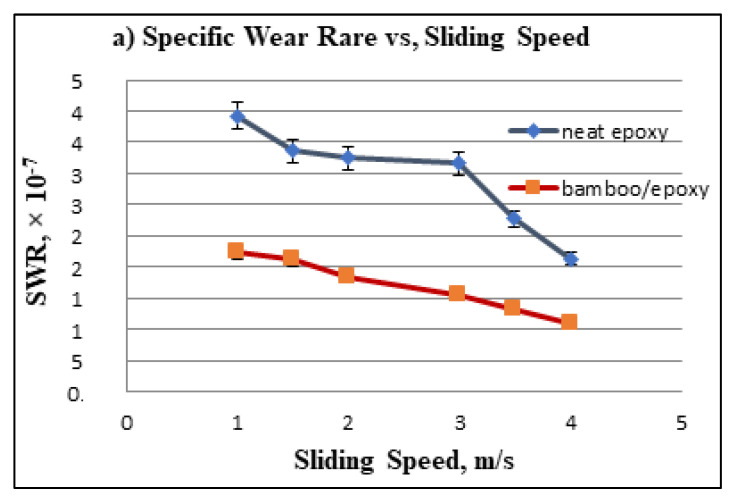

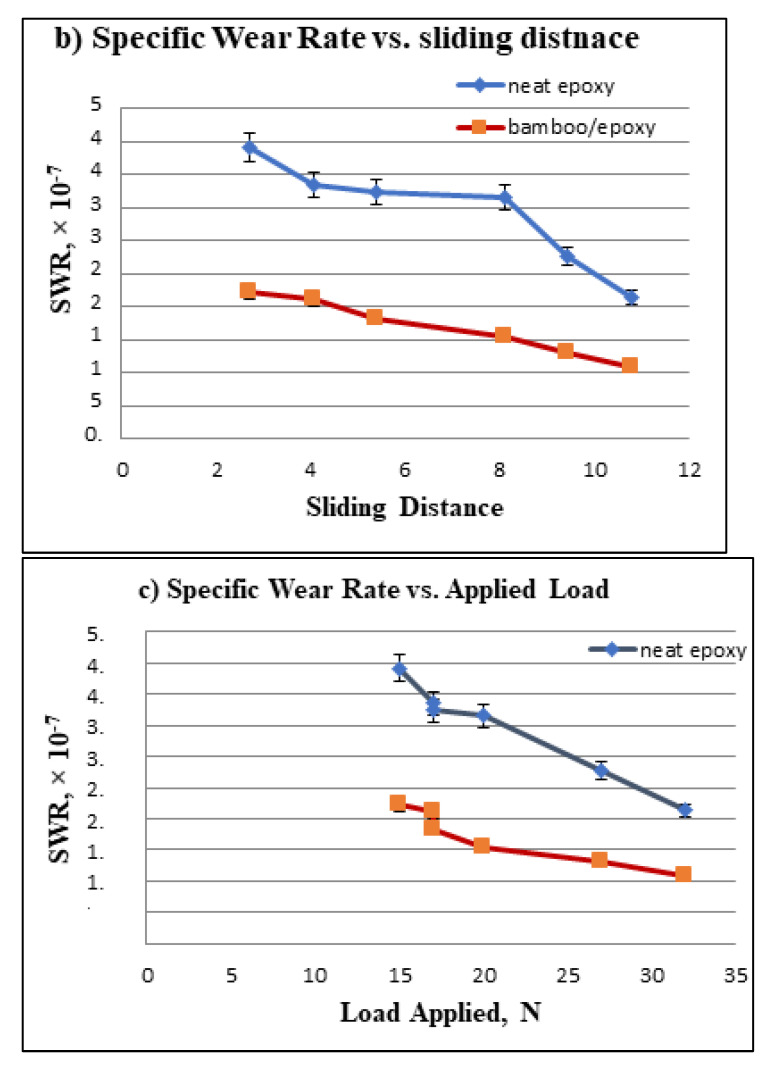
Relationship between specific wear rate (SWR) and sliding distance, sliding speed and applied load.

In addition, the SWR was studied for both NE and S-BFRE using the sliding distance at different counterface sliding velocities subjected to different applied normal loads, as shown in Figure 7. The figure reveals an inverse relationship between the SWR and the sliding distance, the sliding velocity and the applied load (i.e., as the sliding speed, the applied load or the sliding distance increased, the SWR decreased). In detail, the increase in the sliding distance, the applied load and the sliding velocity of the S-BFRE composite yielded an SWR lower than that of NE, as shown in Figure 7. This result clearly shows that the SWR of the polymer (epoxy) improved because it was reinforced with a natural fibre (bamboo).

## 5. Friction Performance

**Figure 8:**
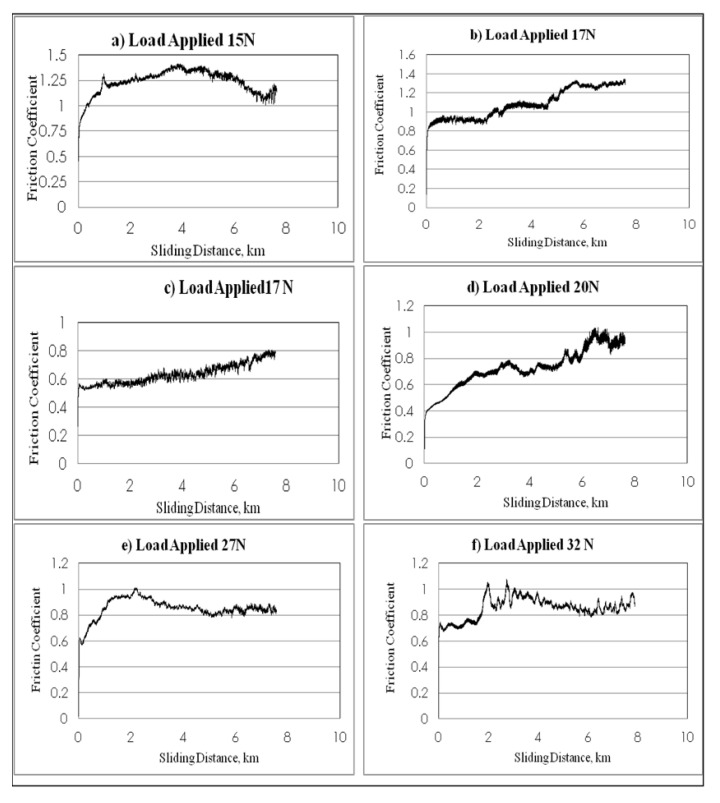
Frictional coefficient of neat epoxy v. sliding distance at different loads and speeds.

The frictional performance of NE and S-BFRE as a function of the sliding distance, the normal applied load and the sliding velocity is plotted in Figure 8. The figure indicates that the friction coefficient for the S-BFRE composite improved on testing it at different sliding velocities, normal applied load and different sliding distances compared with the corresponding results for NE.

In detail, the NE tests showed a gradual increase in frictional values on increasing the sliding distances and sliding velocities. This is because of material lost from the specimen (weight lost) that increased at high sliding speeds. This has led to the possibility that a new material was present between the specimen surface and the counterface; this material calls the adhesive wear. Further, this phenomenon is known as “cold welding” or “rupture” caused by an uneven specimen surface. This new body material led to a coarse contact condition between the counterface and the specimen, which resulted in an increase in the friction coefficient between NE and the counterface, as shown in Figure 8. Conversely, the contact conditions in this test are considered dry conditions because no lubrication was used in this test.

**Figure 9:**
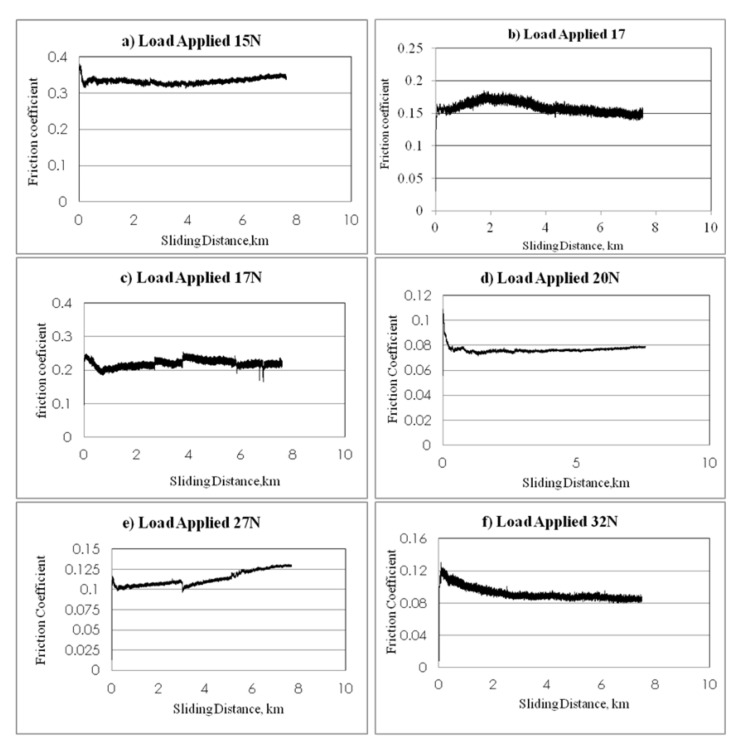
Frictional coefficient of bamboo fibre reinforced epoxy.

For the S-BFRE tests, despite increasing the load applied, the sliding speed and the sliding distance, the frictional values decreased slightly or were steady. This occurred because in this case, the material lost or the third body “adhesive wear” was mixed between the bamboo fibre and the epoxy resin, which affected the contact condition and led to a not fully dry condition (i.e., the adhesive wear contained some of the bamboo fibres, which worked as a self-lubricant and led to steady friction value levels). Thus, this condition helped to keep the frictional values at a steady level or to slightly decrease, as shown in Figure 9.

## 6. Microscope Images

**Figure 10:**
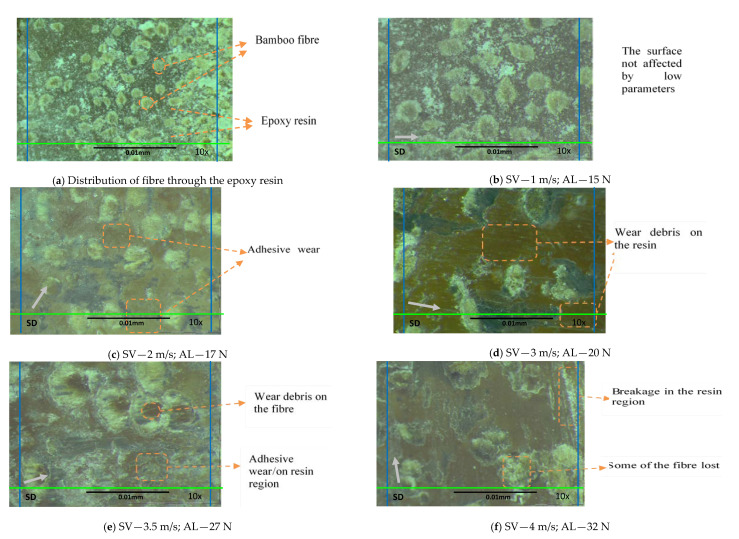
Microscope images showing the morphology of the worn surfaces of the S-BFRE composite test specimen after the tribological test subjected to different operational parameters. Note: SV—Sliding Velocity; AL—Applied Load; SD—Sliding Distance.

The microscope images showing the fibre distribution in the epoxy resin (c.f. Figure 10a) and the morphology of the worn surfaces subjected to different operational parameters are presented in Figure 10, respectively. Figure 10a shows the way that the bamboo fibres were arranged in the epoxy matrix. In addition, a close view indicates that the bamboo fibre was in full contact with the interfacial surface, which helps obtain accurate outcomes.

A close examination of Figure 10b indicates that at 1 m/s sliding velocity and 15 N load, there was a very small deformation in the S-BFRE composite surface, which was associated with the low operational parameter. However, as the sliding velocity and the normal applied load were increased to 2 m/s and to 17 N, respectively, there was adhesive wear associated with wear debris scattered on the resin region, as shown in Figure 10c. However, there seems to have been no damage to the fibrous region (i.e., the operational parameters still had no effect on the bamboo fibre). In contrast, the microscope image clearly revealed that as the sliding speed increased to 3 m/s and the normal applied load to 20 N, adhesive wear, as indicated by wear debris, appeared in the resin region of the tested specimens (see Figure 10d). In addition, Figure 10e indicates evidence of adhesive wear, at 3.5 m/s sliding speed and 27 N normal applied load, on the specimen surface and on the fibrous region as well. The effects of testing at 4 m/s sliding velocity and 32 N normal applied load are illustrated in Figure 10f. The microscope image reveals a tear and a loss of bamboo fibre and a small breakage at the specimen edge. Further, the wear debris is scattered on the resin region as a burned layer. Thus, this image shows the effect of high operational parameters on the S-BFRE composite.

## 7. Roughness Profile

**Figure 11:**
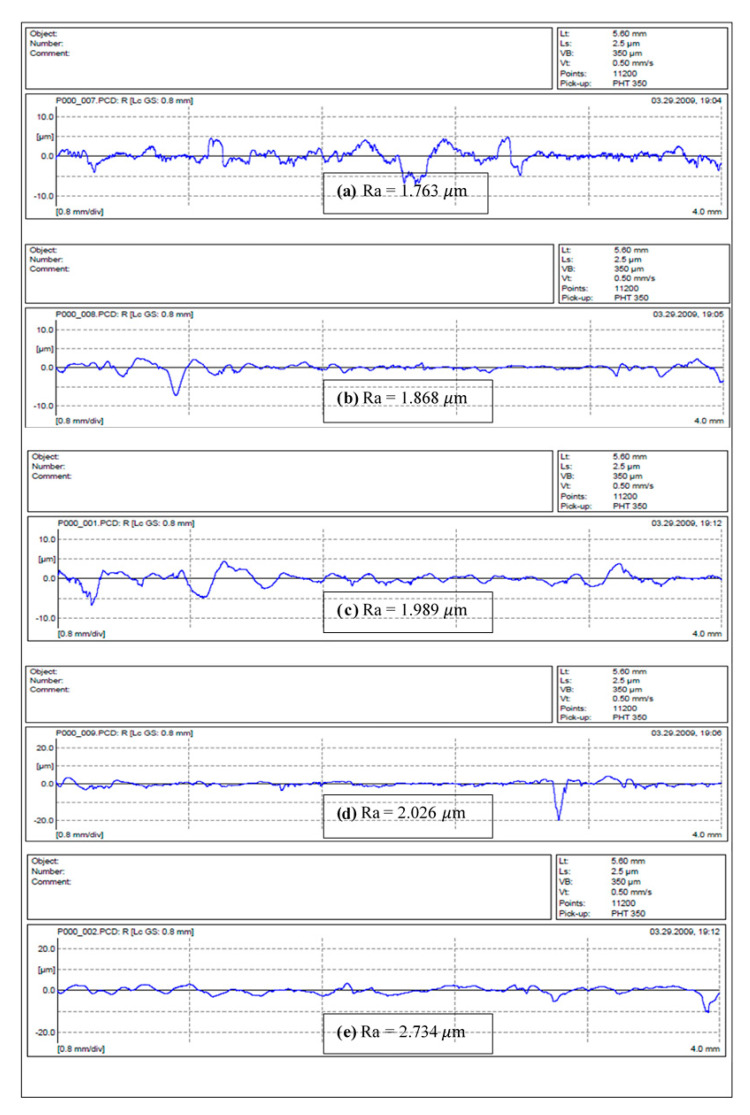
Roughness values of NE samples. Note: SV—Sliding Velocity; AL—Applied Load.

The roughness measurement of sample surfaces after each test is an important parameter that can help to evaluate the experiment results. The Mahr Perthometer was used to measure the roughness profile of all samples. Further evaluation is presented using the roughness profile of selected samples. The results from each test were recorded both in and against the direction of the counterface. In detail, Figure 11 shows that each test yielded different roughness values (i.e., at different sliding distances, speeds and applied loads, the roughness values differed). The values differed because an increase in the applied load led to deeper deformation in the specimen surface, such that very rough surfaces had high roughness values. Further, the roughness values of the S-BFRE composite were significantly higher than those of NE.

### 7.1. Roughness Profile of NE

Figure 11 illustrates the roughness average values of the NE test specimens after the tribological test at different operating parameters. In a nutshell, it can be seen that when the applied load and sliding velocity increases, the Ra values increases as well. It can be said here that the NE samples had experienced thermo mechanical loading namely due to the absence of the bamboo fibres. Hence, when the applied load increases, the samples deform in the direction of the sliding direction causing high material removal rate. Additionally, increasing the sliding velocity further enhanced the material removal process; i.e., fragments of the fracture epoxy resin chips out from the test sample, causing low wear performance of NE. Similar observation was reported by Belal and co-researchers in [6]. The authors claimed that there is a crucial need of reinforcement in neat polymeric resins since the reinforcement elements plays a vital role in absorbing the incoming load to the test specimen and gradually transferring it to the entire specimen. Hence, the material removal rate process can be lowered since the fibre elements are responsible to lock them self firmly with the resin matrix. This can help explain on the high weight loss for the NE test samples during the tribological tests.

### 7.2. Roughness Profile of S-BFRE Composite

**Figure 12:**
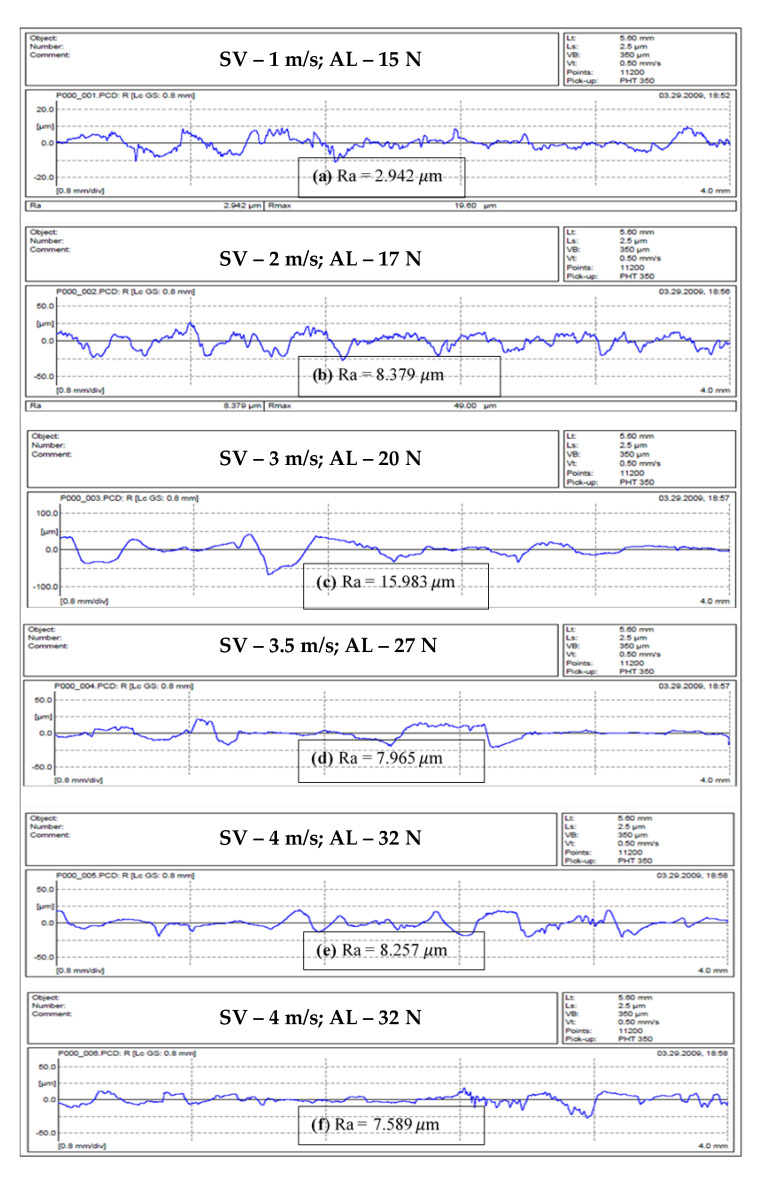
Roughness profile of S-BFRE composite. Note: SV—Sliding Velocity; AL—Applied Load.

The roughness profile of the S-BFRE composite is presented in Figure 12. These were significantly higher than the corresponding NE values. In detail, Figure 12a shows that at the sliding speed of 1 m/s and the applied load of 15 N, the roughness value is 2.942 μm; in contrast, as Figure 11a shows, at the same parameters the NE roughness value is 1.763 μm. Moreover, on increasing the sliding speed to 3.5 m/s and the applied load to 27 N, the roughness value is 8.257 μm for S-BFRE, as shown in Figure 12e, but only 2.734 μm for NE, as shown in Figure 11e. Two potential reasons account for this significant change in the roughness values. The first is that the rigidity of the S-BFRE samples may have increased because the samples were subjected to a high temperature before the tests. Thus, when the composite surface pushed against the counterface, it may have caused higher deformation in the surface areas in contact, which resulted in higher roughness values for the S-BFRE than for the NE. The second reason is that an increase in the sliding velocity or the normal applied load has significant effects on the roughness values (i.e., as the operational parameters increase, the roughness values also increase). These results highlight that factors such as the sliding speed, the applied load and the sliding distance have crucial effects on the roughness profile.

From these results, it can be concluded that the roughness values were higher for S-BFRE than for NE. The first reason for this variation is that the S-BFRE composite had a particular treatment before the tests, and the second is that reinforcing polymer using bamboo fibres improved the polymer strength, resulting in higher roughness values. Further, different operational parameters had entirely different effects on the roughness profile of the two materials.

## 8. Comparison with Previous Related Works

The friction and wear behaviour obtained from the tribology test under dry conditions is explained in this section. In fact, the wear resistance of the S-BFRE composite under different sliding as well as different sliding velocity improved compared with the wear resistance of NE. In addition, the SWR for the S-BFRE composite was better than for the NE, because the effect of thermomechanical loading is a fundamental factor that characterizes the material wear resistance under dry contact conditions.

The friction coefficients for the S-BFRE composite under different dry operational parameters significantly reduced by about 50%, compared with those for the NE. This is because the plastic deformation on the warm surface of the S-BFRE composite was less than that on the NE surface. Further, significant thermomechanical deformation did not occur on the S-BFRE composite surface, which gave the specimen the ability to withstand the applied loads. Recently, several studies have been published about the properties of natural fibre, which helps to understand the behaviour of natural fibres in general. Table 3 presents some of the related works which had been published over the recent years on tribology of polymeric composites involving natural and synthetic fibres.

From Table 5, it can be seen that the result of the current work is quite competitive when compared with glass fibre composites. The SWR and friction coefficient for the S-BFRE composite improved by 13% and 79%, respectively, when compared to glass fibre composite. In a work done by Nirmal and co-researchers [2], bamboo fibres were used as potential reinforcements in mat form with epoxy resin. The work focused on the fibre orientations with respect to the contact condition at the rubbing zone of the BFRE composite / stainless steel counterface; AP, P and N orientations of the bamboo fibre mats. As a result, AP-O demonstrated the best wear performance of the composite when compared to P-O and N-O. With the tribological results for AP-O being compared with the current work (i.e., test performed using a POD setup at SV: 2.83 m/s at AL: 30 N), it is observed that the SWR and friction coefficient improved by 69% and 79%, respectively. Hence, it can be interpreted here that the experimental test parameters such as SV and AL influence the tribological performance of the S-BFRE composite. On the other hand, the distribution of fibres in the resin matrix subjected to the contacting stainless steel counterface plays a crucial role in influencing the wear performance of the test samples. It is to be reminded here that the current work adopted bamboo fibres in ‘stalked’ form during fabrication of the composite, c.f. Figure 3. Hence, the fibres assisted in lowering the material removal process of epoxy resin since the surface wettability of the fibres and resin was high, i.e., no evidence of fibre debonding and fibre pull-out at the microscopy images of the worn samples. This could explain the improved wear and friction performance of the current work when compared to [2].

All in all, the use of natural fibre composites in industries are gaining interest because of their excellent tribological characteristics. For an instance, S-BFRE composite may be used in various applications such as bearings, sliders and bushings. Moreover, its unique advantages, such as low wear resistance, low friction coefficient, light weight, low machining cost, renewability and biodegradability encourage the engineering discipline to adopt this material for future projects. The main challenges faced when using natural fibres in composites materials is that their moisture content within the fibre’s cell wall. Although the fibre is the same, but their moisture content greatly differs when there are harvested at different geographical locations throughout the world. This is a challenge faced by the researchers and scientists in developing high performance composite materials involving natural fibres; i.e., the idea of having low moisture content as a result of suitable fibre chemical treatment prior to composite fabrication. However, more research pertaining the real time application of the S-BFRE composite to the proposed applications above need to be investigated in future thereby opening new research pathways on the subject of interest.

## 9. Conclusions

In conclusion, the adhesive wear of polymer improved following the reinforcement of natural fibre into the polymer matrix (i.e., both the wear lost and the specific wear rate decreased). There was significant reduction in the friction coefficient for the epoxy reinforced by bamboo fibres. This occurred because the distribution of the natural fibre (bamboo) in the polymer matrix helped to reduce the friction between the counterface and the specimen surface (i.e., the bamboo worked as a self-lubricant). The mechanical properties of the composite were improved because it was reinforced with bamboo fibre, and, in turn, the composite could withstand heavier loads. Plastic deformation of the worn surface of the composite was observed at a low sliding velocity and a low applied load. However, there was a breakage in the resin region of the S-BFRE composite because the specimen was subjected to higher operational parameters, which has a significant effect on the wear resistance behaviour. The roughness values of the S-BFRE composite were affected by the operational parameters (i.e., the roughness profile varied as the sliding velocities as well as the applied loads changed).

## Figures and Tables

**Table 2:** Some important information on chemical and physical properties of bamboo fibres [21,22].

Cellulose (%)	Lignin (%)	Pentosans (%)	Ash (%)	Tensile Strength (MPa)	Density (g/cm^3^)
22–50	18–35	12–25	1.5–5.9	110–260	0.4–2.0

**Table 3:** Some important information on single fibre pullout test of bamboo fibres, interfacial shear strength and flexural strength of bamboo fibres/epoxy resin [23,24].

Single Fibre Pullout Test at 6% NaOH Fibre Treatment			
Tensile (MPa)	Young Modulus (GPa)		Strain at Break (%)
363 ± 100	11.2 ± 2.5		3.3 ± 0.5
**Interfacial Shear Strength of Bamboo Fibres/Epoxy Resin**			
42.5 ± 10 MPa at 6 wt% of fibre loading conditions with 6% NaOH treatment			
**Flexural Strength of Bamboo Fibres (length of 15 mm)/Epoxy Resin at 30 wt% of Fibre Loading Conditions with 6% NaOH Treatment**			
**Flexural Modulus (GPa)**		**Flexural Strength (MPa)**	
5.35 ± 0.5		70.6 ± 5	

**Table 4:** Operational parameters selected.

Specimen No	Load (N)	Sliding Speed (m/s)	Sliding Distance (km)
1	10	1	2.5
2	15	1	4.0
3	17	2	5.5
4	20	2	8.0
5	27	3	9.5
6	32	4	11.5

**Table 5:** Comparison of specific wear rate (SWR) and friction coefficient of various natural fibres and synthetic fibres reinforced polymeric composites with the bamboo fibre reinforced epoxy (S-BFRE) composite under dry sliding conditions.

Fibre/Resin	Fibre Treatment	Experimental Parameters	Friction Coefficient; (μ)	Specific Wear Rate (SWR); (mm^3^/N.m)
Bamboo/Epoxy (current work)	6% NaOH	*AL* = 17 N *SV* = 1.5–3 m/s	0.15	16 × 10^−5^
**Kenaf/Epoxy** [25]	None	*AL* = 30 N *SV* = 2.8 m/s	N-O = 0.52 P-O = 0.35 AP-O = 0.38	N-O = 1 × 10^−5^ P-O = 5.5 × 10^−5^ AP-O = 2.5 × 10^−5^
**Seed** **oil palm/Polyester** [26]	None	*AL* = 20 N *SV* = 2.8 m/s	0.68	6 × 10^−5^
**Carbon-Aramid/Epoxy** [27]	None	*pv* = 0.25–1.5 MPa.m/s	0.44 ± 0.05	0.6–1 × 10^−6^
**Glass/Epoxy** [27]	None	*pv* = 0.25–1.5 MPa.m/s	0.7 ± 06	6.5–18 × 10^−6^
**Oil palm/Polyester** [8]	6% NaOH	*AL* = 50 N *SV* = 2.8 m/s	0.35	1.1 × 10^−5^
**Woven Glass/Polyester** [5,28]	None	*AL* = 20 N *SV* = 2.8 m/s	0.45	15 × 10^−5^
**Betelnut/Polyester** [29]	6% NaOH	*AL* = 30 N *SV* = 2.8 m/s	N-O = 0.65 P-O = 0.51 AP-O = 0.49	N-O = 60 ± 4.9 × 10^−5^ P-O = 15 ± 0.5× 10^−8^ AP-O = 5 ± 0.01 × 10^−8^

Note: AP-O—Anti-Parallel Orientation; P-O—Parallel Orientation; N-O—Normal Orientation; pv—pressure velocity; AL—Applied Load; SV—Sliding Velocity

## Data Availability

All the experimental data was obtained through a BOD wear test rig which was build based on ASTM G99-05 standard. The data was captured by the machine which is located at Faculty of Health, Engineering and Sciences, the University Southern Queensland, QLD4350, Toowoomba, Australia.
